# Oral health-related quality of life and loneliness among older adults

**DOI:** 10.1007/s10433-016-0392-1

**Published:** 2016-07-18

**Authors:** Patrick Rouxel, Anja Heilmann, Panayotes Demakakos, Jun Aida, Georgios Tsakos, Richard G. Watt

**Affiliations:** 1 UCL Eastman Dental Institute, 256 Gray’s Inn Road, London, WC1X 8LD UK; 2 UCL, Epidemiology and Public Health, 1-19 Torrington Place, London, WC1E 6BT UK; 3 Division of International and Community Oral Health, Tohoku University, 4-1 Seiryo-machi, Sendai, Japan

**Keywords:** Oral health, Edentate, Quality of life, Loneliness, Depression, Social capital

## Abstract

Loneliness is a serious concern in aging populations. The key risk factors include poor health, depression, poor material circumstances, and low social participation and social support. Oral disease and tooth loss have a significant negative impact on the quality of life and well-being of older adults. However, there is a lack of studies relating oral health to loneliness. This study investigated the association between oral health-related quality of life (through the use of the oral impact on daily performances—OIDP—measure) and loneliness amongst older adults living in England. Data from respondents aged 50 and older from the third (2006–2007) and fifth (2010–2011) waves of the English Longitudinal Study of Ageing were analyzed. In the cross-sectional logistic regression model that adjusted for socio-demographic, socio-economic, health, and psychosocial factors, the odds of loneliness were 1.48 (1.16–1.88; *p* < 0.01) higher amongst those who reported at least one oral impact compared to those with no oral impact. Similarly, in the fully adjusted longitudinal model, respondents who reported an incident oral impact were 1.56 times (1.09–2.25; *p* < 0.05) more likely to become lonely. The association between oral health-related quality of life and loneliness was attenuated after adjusting for depressive symptoms, low social participation, and social support. Oral health-related quality of life was identified as an independent risk factor for loneliness amongst older adults. Maintaining good oral health in older age may be a protective factor against loneliness.

**Electronic supplementary material:**

The online version of this article (doi:10.1007/s10433-016-0392-1) contains supplementary material, which is available to authorized users.

## Introduction

Loneliness can affect people at any stage of life but older people, especially those over 80 years, are at particular risk (Dykstra 2009). International comparative studies have estimated that between 20–30 % of people aged 45 to 79-year olds report moderate or serious loneliness, but amongst those over 80 years the rates of loneliness can reach 40–50 % (Dykstra 2009). Older people are at increased risk of experiencing social isolation through the loss of a spouse, close relatives, and friends. Loneliness needs to be differentiated from social isolation as the latter refers to the absence of relationships with other people (de Jong Gierveld and Havens 2004), in contrast to the subjective nature of loneliness, which refers to the perception of a discrepancy between expectations and satisfaction in a person’s social relationships (Peplau and Perlman 1982). In other words, someone can feel lonely in a crowded room, although they are clearly not socially isolated. Loneliness can be experienced as emotional loneliness—missing the companionship of one particular person such as a spouse or close friend. In contrast, social loneliness refers to the lack of a wider circle of friends and acquaintances that can provide a sense of belonging and companionship.

Additional factors associated with an increased risk of experiencing loneliness include families living at a greater distance from each other, and less cohesive communities (Dykstra 2009; Fokkema et al. 2012; Victor et al. 2005). Deteriorating health may affect an individuals’ ability to maintain their daily lifestyles, including their social participation (Li and Ferraro 2006). The loss of an intimate relationship through widowhood or divorce may also result in feelings of loneliness (Dykstra and Fokkema 2007; Savikko et al. 2005). Therefore, loneliness is clearly related to a lack of diversity in social contacts and family ties (Litwin and Shiovitz-Ezra 2011), and is also related to dissatisfaction with social networks or levels of social support (Tiikkainen and Heikkinen 2005). Structural factors such as low income, education, and living alone or in a residential home can also contribute to loneliness in older people (Hawkley et al. 2008; Savikko et al. 2005).

Poor health is a determinant, as well as a potential consequence of loneliness. Amongst older adults, loneliness has been associated with poor self-rated health (Nummela et al. 2011), limiting long-standing illness and impaired mobility (Steptoe et al. 2013). It is also a significant risk factor for depressive symptoms (Cacioppo et al. 2006; Fokkema et al. 2012), blood pressure (Hawkley et al. 2010), cognitive decline (Boss et al. 2015) and poses a substantial mortality risk (Holt-Lundstad et al. 2010; Steptoe et al. 2013).

One of the key limitations of the literature on loneliness amongst older adults is the lack of studies relating loneliness to oral health. Oral diseases and tooth loss have a significant negative impact on the quality of life and well-being of older adults, with functional, psychological, and social consequences (Gerritsen et al. 2010; Hassel et al. 2011; Hugo et al. 2009). Good oral health is important for social interaction (Donnelly and MacEntee 2012; Tsakos et al. 2013) and general well-being (Hugo et al. 2009). Oral health is a dynamic phenomenon influenced by many factors that change over time, and can generate positive, as well as negative emotions (Brondani et al. 2007). Older adults are often vulnerable, and may require help in maintaining their independence and preserving their confidence in oral health functioning, including daily life activities such as eating, talking and smiling (Chalmers 2003). Even amongst independently living older adults, a number of studies not only have documented the impact of oral health, especially tooth loss, on their quality of life, particularly in terms of difficulty eating but also in terms of social and psychological impacts, such as communication and interaction with other people (Gulcan et al. 2014; Tsakos et al. 2001). The combination of being edentate and having poor oral health functioning could result in increased social isolation and loneliness in later life.

Loneliness has been associated with a decreased likelihood of visiting a dentist (Burr and Lee 2012). Some studies have shown associations between impaired oral health-related quality of life (OHRQoL) and psychological variables like a higher tendency for somatization and depression (Hassel et al. 2011, 2007). A recent study found evidence that a worsening in OHRQoL amongst older adults living in England was associated with increasing levels of depressive symptoms (Rouxel et al. 2016). However, studies on the relationship between OHRQoL and psychological states such as loneliness are sparse. As OHRQoL is a major feature of the daily experiences of older adults, alongside decreased general health and functioning, and shrinking social networks and activities, it is particularly important to examine whether a relationship between OHRQoL and loneliness amongst older adults exists.

There is a body of work that examined demographic characteristics and health as predictors of loneliness, with social networks and social activity as mediating variables (Burholt and Scharf 2014; Creecy et al. 1985; de Jong-Gierveld 1987; Fees et al. 1999). These different predictors of loneliness are included in the Discrepancy Model of Loneliness developed by Perlman and Peplau (1998), which distinguishes between predisposing variables (factors that put people at risk of loneliness but do not necessarily cause it) and precipitating events (factors which lead to a decrease in achieved levels of social interaction). Based upon the analytical model developed by Burholt and Scharf (2014), the predisposing variables are socio-demographic and socio-economic characteristics that are significantly associated with loneliness. In our model, poor oral health is the independent variable and primary precipitating event. We hypothesize that poor oral health will be associated with greater levels of loneliness. We also hypothesize that poor oral health will have a negative influence on social participation, social support and depressive symptoms, which in turn will mediate the association between oral health and loneliness.

This study aimed to examine the cross-sectional and longitudinal associations between OHRQoL and loneliness amongst older adults living in England. In addition, the contribution of socio-demographic and socio-economic factors, health, smoking, and psychosocial factors in explaining the aforementioned association was examined. The study also assessed if the combination of edentulousness with poor OHRQoL increased the risk of loneliness.

## Design and methods

### Data

This study used data from the English Longitudinal Study of Ageing (ELSA)—waves 3 (2006–2007) and 5 (2010–2011). Wave 3 (2006–2007) was the baseline for this study as it was the first wave of ELSA that included oral health measures. The oral health module was not included at wave 4 but was repeated at wave 5.

ELSA is a national cohort study of community-dwelling people aged 50 years and over living in private households in England. The first wave (2002–2003) was drawn from households that participated in the Health Survey for England (HSE) in 1998, 1999, and 2001, and was designed to be representative of the English population. Follow-up interviews took place every two years. Participants completed a face-to-face Computer Assisting Personal Interview (CAPI) and a self-completion questionnaire. Ethical approval for ELSA was given by the London Multi-centre Research Ethics Service and all participants gave their informed consent. The ELSA data, technical details on sampling, and all related documentation can be found at http://www.elsa-project.ac.uk/.

### Outcome variable

In ELSA, loneliness was measured with the three-item short form of the Revised UCLA loneliness scale (Hughes et al. 2004). The three items, ‘How often do you feel you lack companionship?’; ‘How often do you feel left out?’; and ‘How often do you feel isolated from others?’, were rated on three possible responses: ‘Hardly ever or never’, ‘Some of the time’, and ‘Often’. Ratings were summed to produce a loneliness score ranging from 3 to 9, with a higher score indicating greater loneliness. The score was positively skewed, and was therefore dichotomized with those scoring 3–5 classified as ‘not lonely’ and those with a score ≥6 as ‘lonely’ (Steptoe et al. 2013). The three-item scale has a Cronbach alpha coefficient of reliability of 0.83. Previous research has shown strong correlations between the three-item scale and the Revised UCLA scale, as well as convergent and discriminant validity through associations with measures of mood, emotion, and subjective and objective social isolation that are related to loneliness (Hughes et al. 2004).

### Explanatory variable

In ELSA, OHRQoL is measured using a version of the Oral Impacts on Daily Performances (OIDP) questionnaire for elderly populations (Tsakos et al. 2001). The OIDP was developed to assess the oral impacts on the person’s ability to perform daily activities and has demonstrated appropriate psychometric properties in a population-based cross-sectional survey of elderly people in the UK (Tsakos et al. 2001). Participants were asked (yes/no) if they had the following impacts on their daily life due to the condition of their teeth, mouth, and/or dentures: difficulty eating food; difficulty speaking clearly; problems with smiling, laughing, and showing teeth without embarrassment; problems with emotional stability, for example, becoming more easily upset than usual; and problems in enjoying the company of other people such as family, friends, and neighbors. Due to the low prevalence of respondents with difficulties in most of the categories, a dichotomized variable was derived classifying participants reporting at least one oral impact against those reporting none.

### Covariates

Possible covariates were selected based on the results of previous studies identifying the key predictors of loneliness. We included in this study only those that were associated (*p* < 0.05) with both the OIDP and loneliness. Age was categorized into 3 age bands (50–64; 65–74; 75 and over). The cohabiting status was a dichotomous variable, differentiating between those living with a partner/spouse and those who were single. Education was dichotomized into some versus no educational qualifications. Total household wealth (excluding pensions) was calculated using information on financial, physical (such as business wealth, land or jewelry) and housing wealth, minus any debts. For the purpose of this analysis, we used quintiles of total wealth.

A variable indicating the presence of a self-perceived ‘limiting long-standing illness’ was derived from the answers to two questions: whether the participant had any long-standing illness that affected them over a period of time; and if so, whether it limited their activities in any way. Edentulousness was assessed through self-report. Participants were classified as dentate (having some natural teeth) versus edentate (not having any).

The eight-item Centre for Epidemiologic Studies Depression Scale (CES-D) was selected to assess depressive symptomatology in ELSA (Steffick 2000). The item on loneliness was omitted from the CES-D to avoid direct overlap with the loneliness scale (Cacioppo et al. 2010). The binary yes (1) versus no (0) responses were summed to obtain scores ranging from 0 to 7, with higher scores indicating more depressive symptoms. In keeping with previous research using the CES-D instrument (Steptoe et al. 2013), a binary variable was created with respondents reporting three or more symptoms classified as those most at risk of depression. Smoking status was measured using the following categories: never smoked, ex-smoker, and current smoker.

Participants were also asked to indicate whether they were a member of any organization, club or society, and how many committee meetings they attended in a year. A social participation variable with three categories was derived: ‘active member’ (attending at least one meeting in a year), ‘passive member’ (member of at least one organization but did not attend any committee meetings), and ‘not a member’. Social support was assessed by a 3-item scale, asking participants about the emotional support perceived from their spouse/partner, children, other relatives, and friends. The four possible answers were not at all (0), a little (1), some (2), and a lot (3). Items’ scores were summed to obtain a social support scale for all types of relationships combined, ranging from 0 (absolute lack of social support from all sources) to 36 (highest possible score). The derived social support scale was negatively skewed and hence was grouped into tertiles. The Cronbach’s alpha for the social support scale was 0.78.

### Statistical analysis

At ELSA wave three, 8552 non-institutionalized participants completed the interview in person. Of these, 12.7 % (*n* = 1089) did not return the self-completion questionnaire. An additional 14.4 % (*n* = 1072) had missing values on some of the other variables of interest. The rate of missing data was 2.0 % (*n* = 154) for loneliness, 2.8 % (*n* = 213) for wealth, 5.9 % (*n* = 439) for membership in organizations, and 4.5 % (*n* = 337) for social support. Listwise deletion of cases with missing values resulted in a cross-sectional analytical sample of 6391 participants (weighted *N* = 6299). Non-response was higher amongst women, those who were aged 75 years and over, living alone, were less educated, less wealthy, reporting poorer general and oral health, were smokers, not a member of any organization and amongst those reporting to be lonely.

At ELSA wave fifth, 6793 non-institutionalized respondents completed the interviews, out of whom 4943 respondents were part of the wave 3 analytical sample. An additional number of participants were excluded due to missing values in loneliness at wave 5, resulting in a longitudinal sample of 4640 participants.

Descriptive analyses of loneliness by the sample characteristics were performed, with differences between the two loneliness categories being assessed using appropriate statistical tests (*χ* ^2^ or non-parametric tests). We then used logistic regression to analyze the associations between OHRQoL (OIDP) and loneliness while adjusting for covariates. Results are reported as odds ratios (OR) with 95 % confidence intervals (95 %CI). We used interaction terms to examine whether the association between loneliness and OIDP varied by age or gender. None of these interaction terms were statistically significant and for that reason we used the pooled sample. The logistic regression models were sequentially adjusted for age (Model 1), gender and cohabiting status (Model 2), educational qualifications and wealth (Model 3), limiting long-standing illness, depressive symptoms, smoking status, and edentulousness (Model 4), social participation and social support (Model 5). In separate sensitivity analyses, loneliness, CES-D and age were modeled as continuous variables but the associations between loneliness and OIDP remained very similar to those reported in the results. As loneliness had a skewed distribution, it was log-transformed and the scale was inversed and multiplied by −1 to retain the original order of values. The results of the linear regression models were very similar to the logistic regression models, so only the latter models are reported. As edentulousness is common amongst older adults and may affect their eating behavior (Tsakos et al. 2010), a key aspect of OHRQoL, models were also run separately for the dentate and edentate groups.

In addition, for the longitudinal analysis, we created measures of change for the dependent and the main explanatory variables. Responses from both waves were combined to create new variables demonstrating change over time for loneliness and for OHRQoL. The variable of change in loneliness has three categories: no change in loneliness, becoming lonely, and becoming less lonely. The variable of change in OIDP has three categories: no change, incident oral impact, and recovery from oral impact. To explore whether there was any association between changes in loneliness and changes in OHRQoL, multinomial logistic regression was used to estimate the relative risk ratios and 95 % confidence intervals (RRR, 95 %CI) of becoming lonely or becoming less lonely, compared to respondents who did not report any change in loneliness (the reference category). We followed the same pattern of covariate adjustment carried out for the cross-sectional analysis, using baseline values of covariates.

The cross-sectional analyses were carried out using appropriate survey weights, to account for the complex survey design in ELSA as well as non-response at wave 3. Longitudinal weights are provided for ELSA participants who were present at all the ELSA waves. However, the longitudinal analyses did not use the longitudinal weights because a high proportion of the longitudinal sample would have been removed from the analysis. Models were fitted using the Stata/SE 12.1 (StataCorp) software package. This study conforms to the STROBE guidelines.

## Results

Of the 6299 respondents at wave 3, 21.2 % had a high loneliness score, 7.7 % reported at least one oral impact on daily performances in the last six months and 15.4 % were edentate. The characteristics of the analytical sample by loneliness groups are summarized in Table 1. All identified risk factors showed significant associations with loneliness. Loneliness was more prevalent in women, amongst those aged 75 years and over, and amongst those who did not live with a partner. Loneliness was also associated with lower wealth and having no educational qualifications. Chronic health problems such as long-standing limiting illness, reporting 3 or more depressive symptoms and being a smoker, as well as low levels of social participation and social support were also associated with high levels of loneliness.

**Table 1:** Characteristics of the ELSA wave 3 (2006–2007) sample by loneliness: *n* (%) (weighted *N* = 6299)

	Loneliness		
	Low/average *n* = 4963 (78.8 %)	High *n* = 1336 (21.2 %)	*p* value
OIDP			
No oral impact	4652 (80.0 %)	1160 (20.0 %)	
At least one oral impact	311 (63.9 %)	176 (36.1 %)	<0.001
Edentulousness			
Dentate	4257 (79.8 %)	1075 (20.1 %)	
Edentate	706 (73.0 %)	261 (27.0 %)	<0.001
Age group (years)			
50–64	2724 (79.7 %)	696 (20.3 %)	
65–74	1316 (79.8 %)	332 (20.2 %)	
75+	923 (75.0 %)	308 (25.0 %)	0.001
Gender			
Male	2466 (82.2 %)	535 (17.8 %)	
Female	2497 (75.7 %)	801 (24.3 %)	<0.001
Cohabiting status			
Living with partner	3870 (86.4 %)	610 (13.6 %)	
Not living with partner	1093 (60.1 %)	726 (39.9 %)	<0.001
Educational qualifications			
Some qualifications	3602 (80.5 %)	870 (19.5 %)	
No qualification	1361 (75.5 %)	466 (25.5 %)	<0.001
Wealth quintiles			
Wealthiest quintile	1215 (86.5 %)	190 (13.5 %)	
4th	1128 (83.7 %)	220 (16.3 %)	
3rd	976 (78.1 %)	274 (21.9 %)	
2nd	942 (75.8 %)	300 (24.2 %)	
Poorest quintile	702 (66.6 %)	352 (33.4 %)	<0.001
Limiting long-standing illness			
No	3551 (83.3 %)	712 (16.7 %)	
Yes	1412 (69.3 %)	624 (30.6 %)	<0.001
Depressive symptoms ≥3			
No	4368 (85.3 %)	752 (14.7 %)	
Yes	595 (50.5 %)	584 (49.5 %)	<0.001
Smoking status			
Never smoked	1925 (79.9 %)	483 (20.1 %)	
Ex-smoker	2371 (79.9 %)	595 (20.1 %)	
Current smoker	667 (72.1 %)	258 (27.9 %)	<0.001
Social participation			
Active member	1740 (83.5 %)	344 (16.5 %)	
Passive member	1889 (78.4 %)	519 (21.6 %)	
Not a member	1334 (73.8 %)	472 (26.2 %)	<0.001
Social support			
Highest tertile	1789 (93.6 %)	122 (6.4 %)	
Middle tertile	1754 (83.5 %)	347 (16.5 %)	
Lowest tertile	1420 (62.1 %)	867 (37.9 %)	<0.001

*OIDP* oral impacts on daily performances

In the age-adjusted logistic regression model (Table 2, model 1), participants who reported at least one oral impact on their daily life were 2.25 (95 % CI 1.83–2.76) times more likely to report feelings of loneliness compared to those who did not report any oral impact. Further adjustment for gender and cohabiting status (model 2) did not reduce the size of the association. Adjustment for socio-economic factors, including educational qualification and wealth (model 3), had little effect on the association between OIDP and loneliness (OR 2.15; 95 % CI 1.73–2.67). However, when health-related factors were taken into account (model 4), the association was attenuated but still remained statistically significant (OR 1.59; 95 % CI 1.25–2.03). A more detailed analysis (see appendix Table A1) indicated that depressive symptoms contributed the most to the reduction in the odds ratios, from 2.15 (95 % CI 1.73–2.67) to 1.66 (95 % CI 1.30–2.11), whilst smoking status and edentulousness had little effect on the strength of the association between OHRQoL and loneliness. Thus, the variable on depressive symptoms could be either a confounder of the association, or it could potentially be on the pathway between OHRQoL and loneliness. In the fully adjusted model (model 5), accounting for social participation and social support, the size of the association was attenuated further but remained statistically significant (OR 1.48; 95 %CI 1.16–1.89).

**Table 2:** Logistic models of loneliness regressed on OIDP; OR (95 %CI) (weighted *N* = 6299)

OIDP	Loneliness	
	OR (95 % CI)	*p* value
Model 1 (age-adjusted)	2.25 (1.83–2.76)	<0.001
Model 2 (model 1 + socio-demographic factors^a^)	2.23 (1.79–2.77)	<0.001
Model 3 (model 2 + socio-economic factors^b^)	2.15 (1.73–2.67)	<0.001
Model 4 (model 3 + health-related factors^c^)	1.59 (1.25–2.03)	<0.001
Model 5 (model 4 + psychosocial factors^d^)	1.48 (1.16–1.88)	0.001

*OIDP* oral impacts on daily performances

^a^Gender and cohabiting status

^b^Educational qualifications and wealth

^c^Limiting long-standing illness, depressive symptoms, smoking status, and edentulousness

^d^Social participation and social support

In addition, there was little evidence that edentate older adults who reported at least one oral impact were more at risk of loneliness than their dentate peers (see appendix Table A2).

Table 3 summarizes the results of the multinomial logistic regression models of change in loneliness, by change in OHRQoL over the 4-year follow-up period. Between waves 3 and 5, 6.8 % of the respondents reported an incident oral impact and 4.4 % reported a recovery from oral impact. Nearly, 8.4 % of the sample reported becoming lonely at wave 5 and 8.0 % becoming less lonely. The age-adjusted results (model 1) show that respondents with an incident oral impact were significantly more likely to become lonely (RRR 1.76; 95 % CI 1.23–2.50). Following simultaneous adjustment for covariates at wave 3, incident oral impact remained significantly associated with an increased risk of becoming lonely. The association remained practically the same after adjustment for socio-demographic and socio-economic factors (model 3; RRR 1.74; 95 % CI 1.15–2.36). Similar to the cross-sectional results, adjustment for health-related factors (model 4) and psychosocial factors (model 5) attenuated the association, although it remained statistically significant (model 5; RRR 1.56; 95 % CI 1.09–2.25). The risk of becoming lonely was also higher for respondents recovering from oral impact (model 1; RRR 1.62; 95 % CI 1.03–2.52). However, this association became non-significant when adjusting for health-related factors. Change in oral impact was not significantly associated with becoming less lonely.

**Table 3:** Multinomial logistic regression models of change in loneliness by change in OIDPa between Waves 3 (2006–2007) and 5 (2010–2011), *N* = 4640, relative risk ratios (RRRs) 95 % confidence interval (95 % CI)

	Becoming lonely (cases/*n* = 389/4640)			Becoming less lonely (cases/*n* = 372/4640)		
Change in OIDP	No change	Incident oral impact	Recovery from oral impact	No change	Incident oral impact	Recovery from oral impact
Cases/*n*	325/389	40/389	24/389	322/372	29/372	21/372
Model 1 (age-adjusted)	1.00	1.76 (1.23–2.50)**	1.62 (1.03–2.52)*	1.00	1.27 (0.85–1.90)	1.44 (0.90–2.30)
Model 2 (model 1 + socio-demographic factors^a^)	1.00	1.76 (1.23–2.51)**	1.59 (1.02–2.49)*	1.00	1.22 (0.81–1.83)	1.35 (0.84–2.17)
Model 3 (model 2 + socio-economic factors^b^)	1.00	1.74 (1.22–2.48)**	1.55 (1.00–2.42)	1.00	1.20 (0.80–1.81)	1.32 (0.82–2.13)
Model 4 (model 3 + health-related factors^c^)	1.00	1.64 (1.15–2.36)**	1.40 (0.89–2.20)	1.00	1.11 (0.73–1.68)	1.17 (0.72–1.89)
Model 5 (model 4 + psychosocial factors^d^)	1.00	1.56 (1.09–2.25)*	1.34 (0.85–2.11)	1.00	0.98 (0.65–1.49)	1.07 (0.65–1.75)

*OIDP* oral impacts on daily performances

* *p* value < 0.05; ** *p* value < 0.01

^a^Gender and cohabiting status

^b^Educational qualifications and wealth

^c^Limiting long-standing illness, depressive symptoms, smoking status, and edentulousness

^d^Social participation and social support

## Discussion

To the best of our knowledge, this is the first study to assess the association between OHRQoL and loneliness in a nationally representative sample of older adults. Our results have shown a strong and robust association between oral impacts and loneliness both cross-sectionally and longitudinally. The effect sizes from the cross-sectional and longitudinal associations were similar. Older adults with oral impacts had significantly higher risks of being lonely than their counterparts without any oral impacts. This association remained significant after adjustment for age, gender, socio-economic factors, and existing health problems, including depressive symptoms, a major risk factor for loneliness amongst older people (Cacioppo et al. 2006). In addition, edentulousness did not confound the association between OIDP and loneliness, nor did it modify the association. Contrary to the expectation that the combination of edentulousness with poor OHRQoL would have a greater impact on loneliness, it appears that edentulousness and OHRQoL have independent associations with loneliness.

Due to the paucity of research assessing the effect of oral impacts on loneliness, it is difficult to compare our findings with other similar studies. Burr and Lee (2012) demonstrated that loneliness was associated with a reduced likelihood of visiting a dentist. In a US sample of older adults, being widowed (a proxy for poor social relationships) was shown to be associated with lower prevalence of having visited a dentist compared to being married or living with a partner (Watt et al. 2014). Functional measures of social capital (fewer close ties and lower social support) were significantly associated with oral impacts in the same ELSA sample as this study (Rouxel et al. 2015b). However, in a separate longitudinal analysis of ELSA data, poor OHRQoL was not associated with changes in social capital in the ELSA sample of older English adults (Rouxel et al. 2015a).

What are potential pathways linking oral impacts with loneliness? One of the major theoretical explanations of loneliness is through psychosocial predisposing conditions such as poor self-esteem and lack of self-confidence (de Jong Gierveld and Havens 2004; Dykstra 2009). People with low self-esteem and poor self-confidence might be inhibited in their social interactions and feel less attractive to others. The Discrepancy Model of Loneliness (Perlman and Peplau 1998) also highlights the role of precipitating factors such as poor health. Oral impacts including poor eating function, difficulties with speaking and communication and emotional problems may lead to lower self-esteem and reduced self-confidence (Davis et al. 2000). Some recent Japanese studies of older people living with their families showed that those who ate alone were at a particular risk of depressive symptoms (Kuroda et al. 2015; Tani et al. 2015). Our findings provided further evidence for the potential role of the psychosocial pathway, as adjusting for depressive symptoms, social participation and social support partly explained the association between OHRQoL and loneliness.

This study used data from a nationally representative sample of older English adults. Standard measures of OHRQoL and loneliness were used in the analysis and we undertook extensive adjustment of covariates. However, it is important to recognize the limitations of this study. The explanatory variable used was a modified version of the OIDP questionnaire (Tsakos et al. 2001) which may have under estimated the true extent and nature of oral impacts in this sample. Although we adjusted for an extensive range of covariates, residual confounding may still be a matter of concern. Although we used longitudinal data, the observational study design does not allow for a comprehensive assessment of possible causal relationships between oral impacts and loneliness. Indeed, the temporal order may be from loneliness to oral impacts. The 4-year gap between waves 3 and 5 may have been too short to capture significant changes in participants’ oral health or experience of loneliness. There were a small number of respondents whose oral health or loneliness changed between waves 3 and 5. However, despite this limitation, we obtained similar associations in the longitudinal model to the cross-sectional model. In the longitudinal models, recovery from oral impacts was associated with becoming lonely, but this association reduced considerably after adjustment for health-related factors. This suggests that poor health confounds the association between loneliness and recovery from oral impact (respondents who recover tend to have worse health than those who do not change). Thus, the pathway appears to be from health (and oral health) to loneliness, rather than the other way around. Furthermore, the pathways by which loneliness can affect a person’s oral health within a four-year period are unclear. We have based our analyses on the Discrepancy Model of Loneliness (Perlman and Peplau 1998), and have shown that poor oral health can be considered as a precipitating event leading to higher levels of loneliness. Other research also found evidence that an increasing number of chronic conditions led to decreased levels of social interaction (Burholt and Scharf 2014).

## Implications and conclusion

Despite these limitations, the results of this study have numerous implications for gerontologists and practitioners. Researchers in gerontology should consider the impact of poor oral health amongst older adults on their quality of life and well-being. Oral health is an important aspect of health amongst older adults that is often neglected in gerontology research. ELSA is a longitudinal study, and future waves with repeated measures of oral health and loneliness will provide opportunities to fully explore the nature of the relationship between oral impacts and loneliness, and test the potential causal pathways. For dental practitioners and geriatric clinicians, the study emphasizes the importance of maintaining good oral health in later life, as this can have consequences on the wider social life of older adults that goes well beyond their oral health.

In conclusion, the results of this study have shown a strong and consistent association between oral impacts and loneliness in this nationally representative sample of older English adults. The findings were consistent in both cross-sectional and longitudinal analyses, and remained statistically significant after adjusting for a number of factors related to loneliness and OHRQoL. Maintaining good oral health in older age may be a protective factor against loneliness.

## Electronic supplementary material

Below is the link to the electronic supplementary material.

Supplementary material 1 (DOCX 85 kb)
