# Supplementary material for: Oral health-related quality of life and loneliness among older adults
Source: Eur J Ageing. 2016 Jul 18;14(2):101–9. doi: 10.1007/s10433-016-0392-1 (PMC5435788; doi:10.1007/s10433-016-0392-1)
Supplement: Supplementary file 1 — Supplementary material 1 (DOCX 85 kb) [file 10433_2016_392_MOESM1_ESM.docx]

**Supplementary material**

Oral health-related quality of life and loneliness among older adults

European Journal of Ageing

Patrick Rouxel, PhD^1 2^; Anja Heilmann, PhD^2^; Panayotes Demakakos, PhD^2^; Jun Aida, PhD^3^; Georgios Tsakos, PhD^2^; Richard G. Watt, PhD^2^

Patrick Rouxel, ^1^UCL, Eastman Dental Institute, 256 Gray’s Inn Road, London, WC1X 8LD, UK; ^2^UCL, Epidemiology and Public Health, 1-19 Torrington Place, London, WC1E 6BT, UK; [patrick.rouxel@ucl.ac.uk](mailto:patrick.rouxel@ucl.ac.uk)

Anja Heilmann, ^2^UCL, Epidemiology and Public Health, 1-19 Torrington Place, London, WC1E 6BT, UK; [anja.heilmann@ucl.ac.uk](mailto:anja.heilmann@ucl.ac.uk)

Panayotes Demakakos, ^2^UCL, Epidemiology and Public Health, 1-19 Torrington Place, London, WC1E 6BT, UK; [p.demakakos@ucl.ac.uk](mailto:p.demakakos@ucl.ac.uk)

Jun Aida, ^3^Tohoku University, Division of International and Community Oral Health, 4-1 Seiryo-machi, Sendaí Japan; [aidajun@m.tohoku.ac.jp](mailto:aidajun@m.tohoku.ac.jp)

Georgios Tsakos, ^2^UCL, Epidemiology and Public Health, 1-19 Torrington Place, London, WC1E 6BT, UK; [g.tsakos@ucl.ac.uk](mailto:g.tsakos@ucl.ac.uk)

Richard Geddie Watt, ^2^UCL, Epidemiology and Public Health, 1-19 Torrington Place, London, WC1E 6BT, UK; [r.watt@ucl.ac.uk](mailto:r.watt@ucl.ac.uk)

Correspondence should be addressed to P. Rouxel, PhD, UCL Eastman Dental Institute & Research Department of Epidemiology and Public Health, 256 Gray's Inn Road, London, WC1X 8LD, United Kingdom.

Mobile phone: +44 (0) 7527565347

Fax: +44 (0) 20 7813 0280

[patrick.rouxel@ucl.ac.uk](mailto:patrick.rouxel.09@ucl.ac.uk)

**Table A1: Logistic models of loneliness regressed on OIDP^a^; OR (95%CI) (weighted N=6,299)**

|  | Loneliness | |
| --- | --- | --- |
| OIDP | OR (95%CI) | *p-value* |
| Model 1 (age-adjusted) | 2.25 (1.83-2.76) | *<0.001* |
| Model 2 (model 1 + socio-demographic factors^b^) | 2.23 (1.79-2.77) | *<0.001* |
| Model 3 (model 2 + socio-economic factors^c^) | 2.15 (1.73-2.67) | *<0.001* |
| Model 4 (model 3 + health-related factors^d^)  model 3 + limiting long-standing illness  model 3 + depressive symptoms  model 3 + smoking status  model 3 + edentulousness | 1.59 (1.25-2.03)  1.93 (1.55-2.41)  1.66 (1.30-2.11)  2.13 (1.71-2.65)  2.14 (1.72-2.68) | *<0.001*  *<0.001*  *<0.001*  *<0.001*  *<0.001* |
| Model 5 (model 4 + psychosocial factors^e^) | 1.48 (1.16-1.89) | *0.001* |

^a^Oral Impacts on Daily Performances; ^b^gender; cohabiting status; ^c^educational qualification; wealth quintiles; ^d^limiting long-standing illness; depressive symptoms; edentulousness and smoking status; ^e^ social participation and social support

**Table A2: Logistic models of loneliness regressed on OIDP^a^ stratified by dentate and edentate participants; OR (95%CI) (weighted N=6,299)**

|  | Loneliness | | | |
| --- | --- | --- | --- | --- |
|  | Dentate  n=5,331 | | Edentate  n= 967 | |
| OIDP | OR (95%CI) | *p-value* | OR (95%CI) | *p-value* |
| Model 1 age-adjusted 1 | 2.29 (1.82-2.88) | *<0.001* | 1.96 (1.25-3.07) | *0.003* |
| Model 2 (model 1 + socio-demographic factors^b^) | 2.26 (1.78-2.88) | *<0.001* | 2.04 (1.26-3.31) | *0.004* |
| Model 3 (model 2 + socio-economic factors^c^) | 2.15 (1.68-2.75) | *<0.001* | 2.10 (1.30-3.39) | *0.002* |
| Model 4 (model 3+ health-related factors^d^)  *model 3 + limiting long-standing illness*  *model 3 + depressive symptoms*  *model 3 + smoking status* | 1.64 (1.25-2.14)  1.92 (1.49-2.46)  1.72 (1.32-2.25)  2.12 (1.65-2.71) | *<0.001*  *<0.001*  *<0.001*  *<0.001* | 1.38 (0.79-2.41)  1.95 (1.21-3.14)  1.38 (0.79-2.44)  2.11 (1.31-3.40) | *0.150*  *0.006*  *0.153*  *0.002* |
| Model 5 (model 4 + psychosocial factors^e^) | 1.49 (1.14-1.95) | *0.002* | 1.35 (0.78-2.35) | *0.180* |

^a^Oral Impacts on Daily Performances; ^b^gender; cohabiting status; ^c^educational qualification; wealth quintiles; ^d^limiting long-standing illness; depressive symptoms; smoking status; ^e^ social participation and social support
